# Coming together in a rightward direction: post-1980s changing attitudes to the British welfare state

**DOI:** 10.1007/s11135-017-0473-z

**Published:** 2017-03-20

**Authors:** Christopher Deeming, Ron Johnston

**Affiliations:** 1 School of Social Work and Social Policy, University of Strathclyde, Lord Hope Building, Level 6, 141 St James Road, Glasgow, G4 0LT UK; 2 School of Geographical Sciences, University of Bristol, Bristol, BS8 1SS UK

**Keywords:** Welfare, Workfare, Public attitudes, Great Britain, Thatcher, Blair

## Abstract

Major changes in the British welfare state were initiated during the 1980s in response to the 1970s’ stagflation, rapid globalisation and the government’s inability to ensure full employment: the relatively unrestricted payment of unemployment benefits was replaced by a jobseekers’ allowance with applicants obliged to seek work actively and, if required, undergo training. Public support for this shift lagged behind the policy introductions, but from 1997 on there was a major change in attitudes towards welfare beneficiaries. Analysis of social attitude survey data for 1983–2011 shows this change occurred during the decade of relative prosperity under the New Labour governments. There was a growing concentration of anti-welfare attitudes across all social groups, regions and supporters of the main political parties.

## Introduction

The British welfare state was constructed over the first half of the twentieth century, being consolidated by the Labour government elected in 1945 which implemented in large part the principles and mechanisms set out in the 1942 Beveridge report designed to slay the five ‘giant evils’—squalor, want, disease, ignorance and idleness (Timmins 2001). Its nature was widely welcomed across society as a response to the privations of the 1930s depression, the Second World War and the subsequent years of austerity and reconstruction, and its parameters were largely accepted and kept in place by subsequent Conservative governments. Its provisions were extended through the next decades, but came under increasing criticism from some economists, political philosophers and others, leading to increased political conflict over the state’s role in economy and society.

The post-1945 welfare state was built on Keynesian foundations, based on a belief that demand management could ensure a growing economy characterised by full employment, in which welfare state provisions—such as unemployment benefit—catered for those temporarily experiencing want; out-of-work benefits were a stopgap provision for workers between jobs. This system broke down in the 1970s, when the economy was characterised by stagflation and the onset of globalisation. An alternative—Schumpeterian (Jessop 1993)—model of welfare was promoted, notably by New Right think-tanks, and adopted by Conservative politicians (Glennerster and Midgely 1991). The state’s main role of demand management was to be replaced by supply management with a more flexible labour market. Structural changes in the pattern of employment meant that unemployment would be more frequent for many workers, and the state’s role was to minimise that risk through retraining and other policies ensuring that people did not become dependent on welfare benefits (Welshman 2013). The British welfare state was reoriented towards a new welfare-to-work regime that promoted waged work, achieved in part by making unemployment benefit levels decline in real terms—thereby encouraging unemployed workers to move from benefits into paid work.^1^

Welfare reform not only involved a major reorientation of political, economic and social beliefs, but also required a major shift in public attitudes. Without that, support for the new policy orientation would be fragile and—according to the theorists—the country’s economic prosperity threatened. That attitudinal shift was slow to gather pace, with many remaining wedded to the Keynesian principles of full employment guaranteed by the government and a beneficent welfare state for those unable to support themselves through its well-established economic cycles (of so-called ‘boom-and-bust’). But this began to change in the 1990s, as increasing proportions of respondents to inquiries such as the British Social Attitudes annual surveys indicated declining support for the increasingly-expensive system of benefits (see, for example, Hills 2014) on which—or so they were led to believe by the media and some politicians—an increasing proportion of the population were reliant. Living on benefits had become a ‘lifestyle choice’ it was claimed, as more people ‘opted out’ of the workforce.

Although the main parameters of the post-1945 welfare state in Britain were generally accepted, nevertheless—as political and other surveys demonstrated—there was always a divide within society in support for its practices. In general, those most likely to be its beneficiaries—the ‘working class’—were more committed to a high tax and spend policy manifesto, associated with the Labour party, whereas others, while accepting the need for a welfare state safety net, believed that a reduction in benefit entitlements and expenditure, associated with tax cuts, offered a better route to general prosperity. As the latter approach appeared increasingly unsustainable, a new generation of politicians argued that the partisan ideological split had to be healed and removed from the heart of party politics; a new social contract was needed, it was argued, and a new set of post-welfare values was required for success in an economically-globalised world, requiring cross-class acceptance of an alternative regime. How did that happen? Which groups changed most in their attitudes and is there now greater uniformity in popular perceptions of the welfare state and how it should operate? While there has been much work examining attitudes towards welfare in the European context (e.g. Blekesaune and Quadagno 2003; Larsen 2008) as well as more recent work exploring links between social attitudes and the changing political economy of welfare state capitalism in the advanced economies (e.g. Deeming 2016) this article takes an entirely different and original approach, in that it exploits and pools for the first time thirty years of national UK social survey data in order to chart the changes in public opinion across social groups, time and place.

## From the welfare to the workfare state

As part of their response to what they saw as the failings of Keynesian demand management the Thatcher and Major administrations (1979–1997) increasingly adopted policies whose goal was to encourage the unemployed back into work, and the young unemployed—many of whom may not have had previous jobs—to undertake training and other programmes designed to assist their entry into the labour market, the latter through a wide range of job training schemes. By the late 1980s, for example, all claimants of unemployment benefits had to show that they were ‘actively seeking work’. This position was clearly enunciated in a speech by the then Secretary of State for Employment, Norman Tebbit, who—in responding to a claim from within his own party that riots in 1981 were an understandable response to unemployment—stated that “I grew up in the ‘30s with an unemployed father. He didn’t riot. He got on his bike and looked for work, and he kept looking till he found it” (usually referred to as his ‘on your bike’ speech).

But the general public did not appear to respond to this change in government tone and ethos. As Crewe (1988) observes, under the Heath and Wilson/Callaghan governments in the 1970s, public opinion shifted away from Keynesian solutions and moved right, but that opinion moved back again to the left under the Thatcher governments of the 1980s. Such trends appear to broadly conform to the ‘thermostatic model’ of opinion-policy relationships. If trends in public opinion change (i.e. the ‘temperature’ set by the ‘thermostat’ goes up or down) the government will respond to that change and shift policy again (see Soroka and Wlezien 2005). Although Margaret Thatcher won two landslide general election victories in 1983 and 1987 she did not create an enduring rightwards realignment in public opinion regarding welfare reform, and especially attitudes towards out-of-work benefits; the electorate opposed her governments “on the vast array of its specific policy initiatives”. (Indeed, Curtice 1988—argued that a regional divide in attitudes opened up during the first two Thatcher administrations, although it was less substantial than that between the occupational classes.) She had won her elections by default—faced by a Labour party deemed unelectable by many and a split opposition with the rise of the Liberal-SDP Alliance (Crewe and King 1995). Crewe concluded that ‘Much of Thatcherism will die with Thatcher. Its permanent legacy at the level of the mass public will be very limited. It will not have killed off popular socialism, at least not in its welfarist forms. A post-Thatcher Labour government will inherit an electorate as friendly to its major objectives as the 1979 electorate was to those of the Conservatives (Crewe 1989, 250; though see also Dahrendorf 1988).

Although welfare reforms in Thatcher’s third term had created an adult employment training system, modelled on ‘workfare’ in the United States (Peck 2001), that included full-time work done for the unemployment benefit plus an additional £10 top-up, no coherent welfare-to-work regime had been put in place however. Welfare-to-work reform was instead secured by the Labour party under Tony Blair, which while in opposition in 1994 established a Commission on Social Justice: its vision was of a welfare state operating as a “springboard for economic opportunity” rather than a “safety net in times of trouble” (Peck 2001, 276: see also Driver and Martell 1998). Those who could not find employment would be helped into the labour market by public works programmes—with training and, where relevant, childcare provision assisting this transition.

By the time it came to power in 1997, the Blair New Labour government was committed to a workfare regime—generally along lines developed in several States of the USA in the preceding decade and adopted by Bill Clinton before his 1996 re-election campaign (King and Wickham-Jones 1999): as Walker (1998, 35) put it:

Blair’s speeches, and the writings of his close colleagues, resonate with a pot-pourri of US influences … Blair seems to accept that welfare has become a problem rather than a solution, destroying the work ethic and other family values…

Under the Conservatives, the shift towards workfare was in the margins of employment policies; under Blair and New Labour it became the “ideological cornerstone”. As Peck (2001, 262) describes it, Blair’s first post-election-victory speech focused on welfare reform, clearly distancing his ‘New Labour’ project from ‘Old Labour’ by seeking to “reconnect the poor with waged work”—what Blair termed the ‘Welfare to Work programme’. Instead of being claimants of benefits, as in the past, the unemployed would become jobseekers, and a main purpose of the welfare system was to help the unemployed become employable (see Peck 2001, 308ff, and several of the essays in Powell 1999). Blair himself explained the change as “We are … providing people with a ‘hand up’ not a ‘hand out’; previous governments had been satisfied ‘simply to dole out money’ and a senior Cabinet Minister characterised this ‘radical new approach’ as moving “people from being passive recipients of benefits to active jobseekers looking and preparing for work with access to training and job-focused activity” (both quoted in Hills 2014, 4). In future, while people had rights within the welfare state, they also had responsibilities—those rights were conditional on them fulfilling their side of the implicit social contract, which involved them actively seeking work (or undertaking training to assist that process) in order to claim benefits (Griggs and Bennett 2009). Thus Glennerster (2001, 385) concluded that in retrospect Blair’s social policy would be seen as a decisive shift away from Labour’s previous administrations’ positions: as “a move away from an all-inclusive universal welfare state—never achieved in practice but always a dream”—to what he, after Hills and Lelkes (1999), termed ‘selective universalism’. To him—and subsequently to Hills (2014)—this shift in social policy was successful in redistributing income and benefits towards those most in need, but “taking the public with the new strategy will not be easy” (Glennerster 2001, 402).

Workfare programmes were extended under the Conservative-led coalition government that took office in 2010, when the media and political rhetoric became much more aggressive, pitting ‘hard-earning taxpayers’ against those wanting ‘handouts’—‘skivers against strivers’; the ‘strivers’ wanted the welfare state to be fair not only to those who were suffering unemployment and/or poverty as a consequence of the recession but also to themselves, whose cost of living was being squeezed as incomes rose slower than prices and whose taxation was paying for the welfare recipients.^2^ Elements of the media adopted this language (see Jones 2014; Garthwaite 2011; Wiggan 2012); in the *Daily Mail*, for example, Walters and Carlin (2012) presented David Cameron’s decision to remove access to unemployment benefits to those who refuse to seek work as getting behind the workers and cracking down on the shirkers.

## Changing attitudes to unemployment and welfare benefits, 1983–2012

So how widely-shared were these changes in attitudes? Which groups within society changed most, when, where and why? To answer those questions we have assembled relevant survey data covering a period of 30 years.

The data deployed for these analyses have been taken from the suite of attitudinal questions employed in the annual British Social Attitudes’ (BSA) surveys: each question has not been asked every year since the surveys commenced in 1983, but many have been asked in the majority. Three have been selected for analysis here:

- “Around here most people could find a job if they really wanted one”. Respondents were asked if they agreed strongly, agreed, neither agreed not disagreed, disagreed, or disagreed strongly with this statement. As our focus is on the growth in anti-welfare sentiments, we collapsed this into a binary for all respondents—those who agreed or strongly agreed were coded 1 and the remainder (including those who were neutral) were coded 0.
- “Welfare benefits are either too low and cause hardship or too high and discourage work”. Those who agreed with the latter statement were coded 1 and all other respondents were coded 0.
- “If benefits weren’t so generous people would learn to stand on their own two feet”. Those who agreed or agreed strongly with this statement were coded 1 and all other respondents were coded zero.^3^

**Fig. 1:**
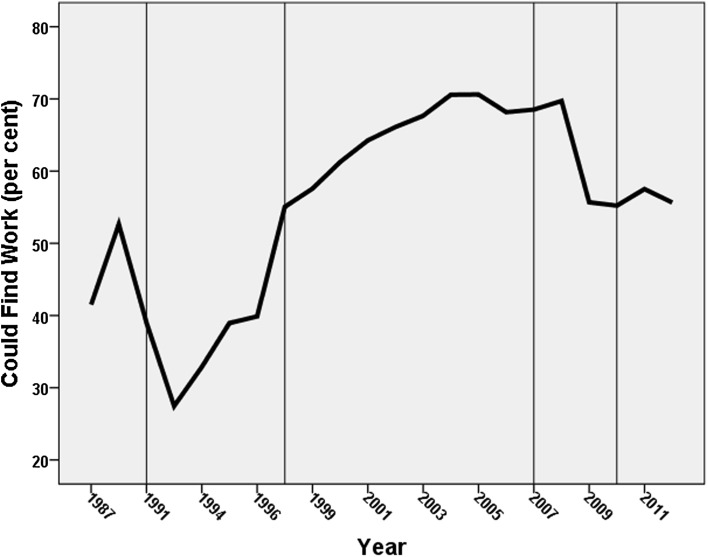
Changes in the percentage agreeing that there were sufficient jobs available locally. (The periods with different Prime Ministers are divided by the *vertical lines*)

**Fig. 2:**
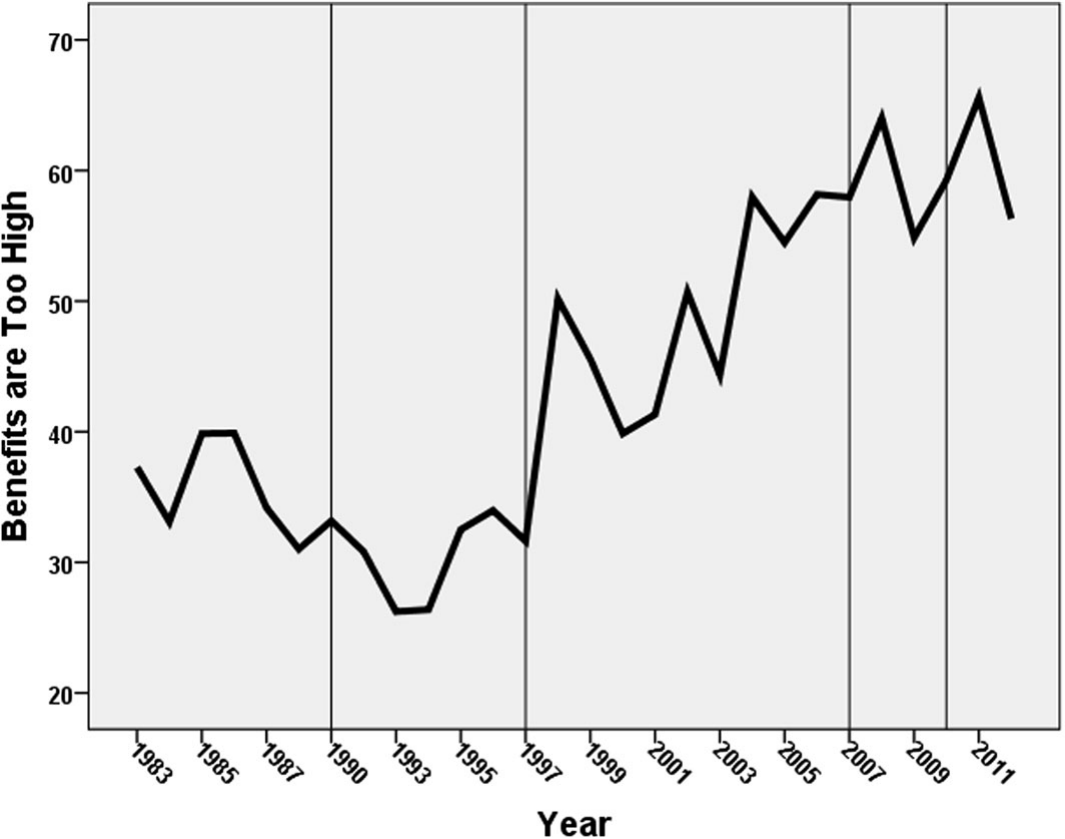
Changes in the percentage agreeing that benefits are too high and discourage work. (The periods with different Prime Ministers are divided by the *vertical lines*)

**Fig. 3:**
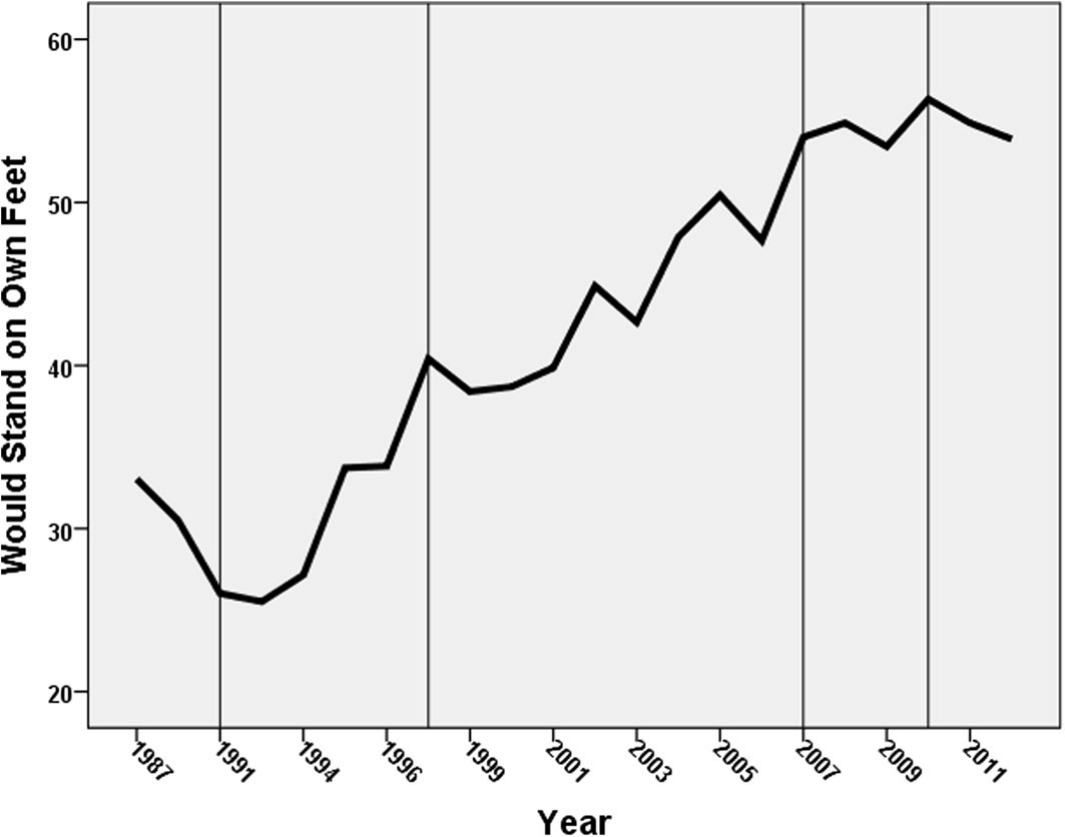
Change in the percentage who agreed that if benefits weren’t so generous people would learn to stand on their own two feet. (The periods with different Prime Ministers are divided by the *vertical lines*)

Figures 1, 2 and 3 show the trends in the percentage of respondents coded 1 on each of these variables, with the sequence divided into five sections according to who was Prime Minster at the time (1983–1990—Margaret Thatcher, Conservative; 1990–1997—John Major, Conservative; 1997–2007—Tony Blair, Labour; 2007–2010—Gordon Brown, Labour; 2010–2012—David Cameron, Conservative leader of a Conservative-Liberal Democrat coalition). There is a common pattern to all three graphs: the percentage of respondents giving an anti-welfare response fell to its lowest levels during the early years of Major’s premiership (i.e. at the beginning of the 1990s), but then increased through the remainder of his term and virtually all of Tony Blair’s. Gordon Brown’s relatively short period of power was marked by stasis on one of the indicators and decline on the other two, and stasis was the main characteristic of the first years of the coalition government led by David Cameron.

From the mid-1990 s on there was a strong trend in British attitudes away from a pro-welfare stance and towards a neo-liberal, ‘workfare not welfare’, position, therefore. On each of the three variables the percentage expressing the latter sentiment more than doubled between the early years of John Major’s premiership and the end of Tony Blair’s. Thenceforth there was little evidence of a return even to the levels of support for the welfare state that characterised the last years of Margaret Thatcher’s administrations although the percentage agreeing that the unemployed in their local area could find work if they wanted to fell considerably after the financial crashes of 2008 following which unemployment increased rapidly.

But did this change in attitudes occur equally across society: given that many studies have shown marked differences across various groups in their attitudes, were there variations among socio-economic and -demographic groups in the extent of the switch? To address that question, we fitted a series of baseline binary logistic regression models to each of the three dependent variables. After several exploratory analyses, we selected ten categorical variables for which there were statistically significant relationships, as follows^4^:

- Age: respondents were grouped into three categories—15–24; 25–64; and over 64—with the former as the comparator;
- Sex: female respondents are contrasted with males;
- Ethnicity: Black and Other Minority Ethnic group members (BME) were contrasted with Whites (including all others);
- Religiosity: those respondents who indicated that they were religious were contrasted with those who were not;
- Trade Union membership: those who were not members were contrasted with those who were;
- Employment Status: those who were either unemployed or not economically active were contrasted with those members of the adult population who were employed (including the self-employed);
- Occupational Class: those whose current or most recent occupation was either in the routine non-manual or manual categories were contrasted with those in the salariat;
- Educational Qualifications: those with either qualifications below degree level or with no qualifications were contrasted with those having degrees;
- Region: those living in either the other eight English regions, or Wales, or Scotland were contrasted with those living in Northeast England (in general, the country’s most disadvantaged region); and
- Period: those living in the years when either Major, Blair, Brown or Cameron was prime minister were contrasted with those living under the Thatcher administration.

The results of these regressions are in Table 1, with for each variable the b coefficient, its standard error (se) and its associated exponent (odds ratio).

**Table 1:** Binary logistic regressions of three anti-welfare indicators

	Jobs for unemployed			Level of benefits			Incentive to work		
	b	se	exp	b	se	exp	b	se	exp
Constant	**–0.15**	0.03		**–0.57**	0.03		–**0.47**	0.03	
Age (comparator—15–24)									
25–64	**–0.22**	0.03	0.80	**–0.10**	0.03	0.90	**–0.09**	0.03	0.91
65+	**–0.13**	0.05	0.88	**0.34**	0.04	1.41	**0.57**	0.05	1.76
Sex (comparator—Male)									
Female	0.03	0.02	1.03	**0.07**	0.02	1.07	**–0.09**	0.02	0.92
Ethnicity (comparator—White)									
BME	**0.16**	0.05	1.17	–**0.09**	0.04	0.91	**0.37**	0.05	1.45
Religiosity (comparator—not religious)									
Religious	**0.07**	0.12	1.07	**0.13**	0.02	1.13	**0.09**	0.02	1.09
Trade union (comparator—Union member)									
Not a member	**0.29**	0.02	1.33	**0.21**	0.02	1.24	**0.23**	0.02	1.26
Employment status (comparator—employed)									
Unemployed	**–1.10**	0.05	0.33	**–1.39**	0.05	0.25	**–1.16**	0.06	0.32
Not economically active	**–0.45**	0.03	0.64	**–0.31**	0.03	0.73	**–0.31**	0.03	0.74
Occupational class (comparator—salariat)									
Other non–manual	**0.10**	0.03	1.10	0.09	0.03	1.01	**0.12**	0.03	1.13
Manual	**0.17**	0.03	1.18	**–0.16**	0.02	0.85	**0.07**	0.03	1.07
Educational qualifications (comparator—degree or higher)									
Less than degree	**0.47**	0.03	1.60	**0.57**	0.03	1.76	**0.60**	0.03	1.82
No qualifications	**0.62**	0.04	1.86	**0.52**	0.03	1.68	**0.83**	0.04	2.29
Region (comparator—Northeast)									
Northwest	**0.21**	0.05	1.23	**0.32**	0.05	1.38	**0.16**	0.05	1.18
Yorkshire/Humber	**0.28**	0.05	1.32	**0.28**	0.04	1.32	**0.21**	0.05	1.23
East Midlands	**0.33**	0.05	1.40	**0.43**	0.05	1.54	**0.30**	0.05	1.35
West Midlands	**0.49**	0.05	1.64	**0.55**	0.04	1.74	**0.34**	0.05	1.40
Southwest	**0.50**	0.05	1.66	**0.60**	0.05	1.82	**0.32**	0.05	1.38
Eastern	**0.59**	0.05	1.80	**0.67**	0.05	2.00	**0.43**	0.05	1.53
London	**0.48**	0.05	1.62	**0.40**	0.04	1.48	**0.29**	0.05	1.35
Southeast	**0.70**	0.04	2.02	**0.56**	0.04	1.75	**0.30**	0.05	1.35
Wales	**0.49**	0.05	1.64	**0.31**	0.05	1.36	**0.22**	0.05	1.24
Scotland	**0.26**	0.05	1.29	**0.31**	0.05	1.36	**0.15**	0.05	1.16
Government (comparator—Thatcher)									
Major	**–0.55**	0.04	0.28	**–0.27**	0.03	0.77	–0.03	0.04	0.98
Blair	**0.72**	0.04	2.06	**0.55**	0.03	1.74	**0.63**	0.04	1.88
Brown	**0.81**	0.05	2.24	**1.12**	0.04	3.07	**1.08**	0.05	2.95
Cameron	**0.38**	0.04	1.46	**1.12**	0.04	3.085	**1.14**	0.05	3.11
N	47,080			59,157			47,061		
Nagelkerke R^2^	0.12			0.12			0.11		
% correctly classified	64.6			62.2			62.4		

Coefficients statistically significant at the 0.05 level or better are shown in bold

A number of general patterns emerge from these regressions. Those aged 25–64 were less likely to be anti-welfare than either young adults or older persons, for example (with an exception for the latter group on the variable relating to the availability of jobs locally). Those who were of a religious disposition were more likely to be anti-welfare than those who were not religious, as were those with lower or no qualifications compared to those with degrees; the unemployed and (to a lesser extent) those outside the labour force were less likely to be anti-welfare, not surprisingly, than those in employment, and trades union members were more likely to be pro-welfare than those who were not.

There was less consistency on some of the other variables, however. Females were more likely than males to believe that the level of benefits was too high, for example, but less likely to consider that lower welfare payments would encourage people to stand on their own two feet; there was no significant difference between the two in their views on the availability of jobs locally.

The blocks of significant coefficients—all of them positive—for regions show that anti-welfare opinions were more common (holding constant all of the other variables) elsewhere in Great Britain than they were in the Northeast of England. Those geographical variations were patterned commensurate with the well-known north:south divide in the British economy, society and polity. Whereas respondents in the other northern English regions (Northwest, and Yorkshire and the Humber) and in Scotland were 20–30 per cent more likely to be anti- than pro-welfare than those in the Northeast (as shown by the exponents), those in England’s southern regions—the economically more-advantaged areas in recent decades—were above 50 per cent more likely to be anti-welfare on the first two of the dependent variables, with London and the Midlands regions between the two groups.

Finally, the variables representing which Prime Minister was in power identify differences consistent with the trends shown in Figs. 1, 2 and 3. Respondents were less anti-welfare during Major’s premiership than they were under Thatcher’s (though the difference was statistically insignificant on the third variable). There was then a substantial increase during the next two premierships—with the anti-welfare levels being between 1.7 and 3.0 times as large under Blair and then Brown respectively as they were under Thatcher, with that difference either levelling-off or, in the case of the jobs availability variable, substantially falling again under Cameron.

## A dividing society?

Most of those differences are not unexpected; they reflect the well-established social divides within British society. But given the major shift towards anti-welfare attitudes from the mid-1990s on, was that change consistent across all groups, or did it vary within and between them? To address that question, we fitted a further series of exploratory models in which all of the independent variables were interacted with the four period variables. The final versions, shown in Table 2, included four of those sets of interactions—with age, trade union membership, occupational class, and educational qualifications—but excluded the others because of insignificant and/or insubstantial findings (these were for sex, ethnicity, religiosity, and employment status: those variables were included in the final model—as was region (see below)—but not their interactions with period). Table 2 shows only the coefficients for those variables that were interacted with period in the final version.

**Table 2:** Binary logistic regressions of three anti-welfare indicators with interaction terms

	Jobs for unemployed			Level of benefits			Incentive to work		
	b	se	exp	b	se	exp	b	se	exp
Age (comparator—15–24)									
25–64	–0.10	0.12	0.91	**0.27**	0.08	1.31	**0.31**	0.14	1.36
65+	0.10	0.15	1.10	**1.01**	0.10	2.75	**1.09**	0.16	3.00
Trade union (comparator—union member)									
Not a member	**0.58**	0.08	1.78	**0.42**	0.06	1.52	**0.33**	0.09	1.40
Occupational class (comparator—salariat)									
Other non-manual	–0.03	0.10	0.98	–0.08	0.07	0.92	–0.04	0.11	0.97
Manual	–0.04	0.10	0.97	**–0.35**	0.07	0.70	–0.14	0.10	0.87
Educational qualifications (comparator—degree or higher)									
Less than degree	**0.93**	0.15	2.54	**0.90**	0.11	2.45	**0.91**	0.18	2.49
No qualifications	**1.07**	0.16	2.91	**0.86**	0.12	2.36	**1.26**	0.19	3.54
Government (comparator—Thatcher)									
Major	–0.06	0.21	0.95	0.20	0.16	1.22	0.22	0.25	1.25
Blair	**1.33**	0.20	3.77	**1.31**	0.14	3.70	**1.31**	0.23	3.69
Brown	**1.49**	0.25	4.42	**2.31**	0.20	10.09	**2.14**	0.27	8.49
Cameron	**1.19**	0.21	3.30	**2.48**	0.17	11.97	**2.14**	0.25	8.53
Interactions									
Government-age									
Major—25–34	–0.29	0.14	0.75	**–0.29**	0.10	0.75	–0.29	0.16	0.75
Blair—25–34	–0.06	0.13	0.95	**–0.47**	0.09	0.63	**–0.46**	0.15	0.53
Brown—25–34	–0.04	0.18	0.96	**–0.62**	0.14	0.54	**–0.64**	0.19	0.63
Cameron—25–34	–0.15	0.15	0.86	**–0.57**	0.12	0.57	**–0.46**	0.15	0.53
Major—64+	**–0.36**	0.17	0.70	**–0.49**	0.13	0.61	–0.30	0.18	0.74
Blair—64+	–0.13	0.16	0.88	**–0.79**	0.11	0.46	**–0.54**	0.17	0.58
Brown—64+	–0.26	0.21	0.77	**–1.01**	0.18	0.37	**–0.94**	0.22	0.39
Cameron—64+	**–0.39**	0.18	0.68	**–1.04**	0.14	0.36	**–0.86**	0.19	0.42
Government-trade union									
Major—not a member	**–0.27**	0.09	0.76	–0.08	0.07	0.93	–0.05	0.10	0.96
Blair—not a member	**–0.36**	0.09	0.70	**–0.26**	0.06	0.57	–0.11	0.10	0.90
Brown—not a member	**–0.37**	0.12	0.69	**–0.56**	0.11	0.77	**–0.26**	0.13	0.77
Cameron—not a member	**–0.23**	0.10	0.80	**–0.33**	0.08	0.72	–0.15	0.11	0.86
Government–occupational class									
Major—other nonmanual	0.03	0.12	1.03	0.05	0.09	1.06	0.15	0.12	1.16
Blair—other nonmanual	0.17	0.11	1.18	0.14	0.08	1.15	0.17	0.12	1.19
Brown—other nonmanual	0.27	0.15	1.30	0.18	0.12	1.20	0.24	0.15	1.28
Cameron—other nonmanual	0.09	0.12	1.10	–0.01	0.10	0.99	0.14	0.13	1.45
Major—manual	0.17	0.11	1.19	0.14	0.08	1.15	**0.25**	0.12	1.29
Blair—manual	**0.23**	0.10	1.26	0.24	0.07	1.27	**0.24**	0.11	1.27
Brown—manual	**0.29**	0.14	1.34	**0.32**	0.12	1.38	0.23	0.14	1.25
Cameron—manual	0.17	0.12	1.19	0.16	0.09	1.17	0.07	0.12	1.07
Government-educational qualifications									
Major—below degree	–0.13	0.17	0.88	–0.25	0.13	0.78	–0.06	0.20	0.95
Blair—below degree	**–0.47**	0.16	0.62	**–0.24**	0.12	0.79	–0.27	0.19	0.77
Brown—below degree	**–0.60**	0.18	0.55	**–0.32**	0.15	0.73	–0.37	0.21	0.69
Cameron—below degree	**–0.68**	0.16	0.51	**–0.69**	0.13	0.50	**–0.51**	0.19	0.60
Major—no qualifications	–0.11	0.19	0.90	–0.18	0.15	0.84	–0.18	0.22	0.83
Blair—no qualifications	**–0.46**	0.17	0.63	**–0.26**	0.13	0.77	–0.42	0.24	0.65
Brown—no qualifications	**–0.58**	0.22	0.56	**–0.48**	0.18	0.62	**–0.50**	0.20	0.61
Cameron—no qualifications	**–0.62**	0.18	0.52	**–0.80**	0.15	0.45	**–0.61**	0.21	0.54
N	47,080			59,157			47,061		
Nagelkerke R^2^	0.13			0.12			0.11		
% correctly classified	64.7			62.7			62.4		

Coefficients statistically significant at the 0.05 level or better are shown in bold

With the first independent variable—age—there is little patterning for the first anti-welfare variable (the availability of jobs locally) but substantial differences on the other two. For example, the block of coefficients for the ‘raw’ variable shows that in the first period those aged 25–64 and, even more so, those aged over 64 were much more likely to say that benefit levels were too high and that if they were lower people would have a greater incentive to stand on their own two feet, than were those who were under 25: British people became more anti-welfare as they aged, it seems. But the significant negative coefficients for the interaction variables show that the later the period the narrower that gap between the two older age groups and the under-25s: there was a convergence in anti-welfare attitudes over time. The same is true with the patterns for trade union membership; in the first period non-members were substantially more likely to be anti-welfare on all three indicators than were members (exponents of 1.78, 1.52 and 1.40 respectively). But negative coefficients for the interaction variables—especially on the first two indicators—again show those differences narrowing over time. A very similar patterning occurs with the educational qualifications variable: those lacking qualifications were more likely to be anti-welfare than those with degrees, for example, but that difference is reduced across the three decades: again, convergence across the groups over time. Only with the occupational classes is there a less clear-cut situation: where the coefficients are significant, they suggest that manual workers became more anti-welfare in the middle periods.

That shift resulted from movement away from the pro-welfare stance over time, rather than a movement of those who were initially anti-welfare in the other direction. This is illustrated in Table 3 using one of the questions—whether benefits are so high that they discourage work—for the three variables with significant interaction coefficients in Table 2. The gap between the youngest and the oldest group was 25 points during the Thatcher premiership, with the oldest the least pro-welfare. It halved during Major’s term as Prime Minister, halved again under Blair, and was negligible for the last two periods. The main shift was in the attitudes of the younger groups: between the periods the percentage of those aged 15–24 sharing the anti-welfare attitude increased by 152 per cent, whereas for those aged 25–64 the increase was 69 per cent and for those aged 65 and over just 28 per cent. The young were won over to attitudes already held by a majority of the old. The next block shows that the main change in answers to that question was for trades unionists, with no difference between them and non-members in their attitude to benefit levels in the final period. And finally, the difference between those with degrees and those without any qualifications halved between Thatcher’s and Cameron’s premierships, with the change in former group’s percentage thinking benefit levels were too high being twice that of the latter. In all three groups, therefore, the change was in the same direction: those who expressed pro-welfare positions—who initially believed benefits were too low—moved towards those who were anti-welfare, with no substantial counter-movement in the other direction.

**Table 3:** Variations in the percentages agreeing that benefits are too high and discourage work, by period

	Thatcher	Major	Blair	Brown	Cameron
Age group					
15–24	25	27	51	66	63
25–64	35	29	47	60	59
65+	50	39	57	66	64
Δ	25	12	6	0	1
Trade union					
Member	30	26	47	65	61
Not a member	38	32	50	61	61
Δ	8	6	3	4	0
Qualifications					
Degree	24	20	38	55	60
Below degree	37	31	52	66	63
None	37	32	50	58	66
Δ	13	12	12	3	6

The Δ rows show the difference between the percentage in the top and bottom rows for that variable

## A changing geography

A further set of analyses focused on the regional variables alone, exploring whether the country became more or less divided spatially over the period. (Support for the country’s main political parties showed a pronounced spatial divide during the 1980s, that narrowed in the 1990s but widened again from 2001 on: Johnston et al. 1988, 2005.) For this, all of the other variables were retained in the models but the number of regions was reduced to six, based on the pattern of coefficients in Table 1: North (Northeast, Northwest, and Yorkshire and the Humber); Midlands (East and West); South (Eastern, Southeast and Southwest); London; Wales; and Scotland. The relevant coefficients for those six regions, the five periods, and the interactions between those two groups are in Table 4; the coefficients for the other variables retained from the previous models are not reported again to save space. The first two blocks of coefficients show the same trends as in Table 1: an increase in anti-welfare sentiment over time, and more anti-welfare sentiment in all regions except Scotland when compared to the three northern regions.

**Table 4:** Binary logistic regressions of three anti-welfare indicators against region, with interactions

	Jobs for unemployed			Level of benefits			Incentive to work		
	b	se	exp	b	se	exp	b	se	exp
Constant	**–6.16**	0.82		**–3.60**	0.50		**1.73**	0.85	
Government (comparator—Thatcher)									
Major	–0.13	0.08	0.88	–0.17	0.05	0.85	–0.07	0.08	0.94
Blair	**1.21**	0.07	3.35	**0.87**	0.05	2.39	**0.70**	0.08	2.02
Brown	**1.38**	0.10	3.96	**1.31**	0.07	3.70	**1.09**	0.10	2.98
Cameron	**0.92**	0.08	2.52	**1.38**	0.06	4.00	**1.15**	0.08	3.17
Region (comparator—North)									
Midlands	**0.60**	0.10	1.82	**0.42**	0.06	1.53	**0.33**	0.11	1.39
London	**1.00**	0.13	2.73	**0.84**	0.08	2.31	**0.50**	0.13	1.65
South	**1.37**	0.09	3.93	**0.77**	0.05	2.17	**0.35**	0.10	1.42
Wales	**1.25**	0.13	3.49	**0.49**	0.07	1.63	0.15	0.13	1.16
Scotland	0.12	0.16	1.12	0.14	0.09	1.15	0.07	0.17	1.07
Interactions									
Major—Midlands	**–0.26**	0.12	0.78	–0.10	0.08	0.90	–0.17	0.12	0.84
Blair—Midlands	**–0.35**	0.11	0.70	**–0.17**	0.07	0.85	–0.12	0.11	0.89
Brown—Midlands	**–0.56**	0.15	0.57	0.06	0.12	1.06	–0.02	0.15	0.98
Cameron—Midlands	**–0.46**	0.12	0.63	**–0.26**	0.09	0.78	–0.10	0.13	0.91
Major—London	**–0.57**	0.15	0.57	–0.19	0.10	0.83	–0.04	0.15	0.96
Blair—London	**–0.84**	0.14	0.43	**–0.89**	0.09	0.41	**–0.43**	0.14	0.65
Brown—London	**–0.89**	0.18	0.41	**1.11**	0.13	0.33	**–0.66**	0.18	0.51
Cameron—London	**–0.87**	0.15	0.42	**–1.03**	0.10	0.36	**–0.51**	0.15	0.60
Major—South	**–0.84**	0.11	0.43	**–0.14**	0.07	0.87	0.02	0.11	1.02
Blair—South	**–0.97**	0.10	0.38	**–0.48**	0.06	0.62	**–0.19**	0.10	0.82
Brown—South	**–1.15**	0.13	0.32	**–0.30**	0.10	0.74	–0.13	0.13	0.88
Cameron—South	**–1.12**	0.11	0.33	**–0.45**	0.08	0.64	–0.17	0.11	0.84
Major—Wales	**–0.83**	0.14	0.44	–0.19	0.10	0.83	–0.02	0.15	0.98
Blair—Wales	**–1.13**	0.14	0.33	**–0.60**	0.08	0.55	–0.09	0.14	0.91
Brown—Wales	**–1.16**	0.21	0.31	**–0.40**	0.17	0.67	0.16	0.21	1.17
Cameron—Wales	**–1.26**	0.17	0.29	**–0.68**	0.13	0.51	–0.16	0.17	0.85
Major—Scotland	0.20	0.18	1.22	0.05	0.13	1.05	0.18	0.19	1.20
Blair—Scotland	–0.11	0.17	0.90	–0.07	0.10	0.94	–0.05	0.18	0.95
Brown—Scotland	–0.19	0.21	0.83	–0.14	0.15	0.87	–0.18	0.21	0.84
Cameron—Scotland	–0.20	0.18	0.82	**–0.32**	0.13	0.72	–0.33	0.19	0.72
N	54,747			71,830			54,732		
Nagelkerke R^2^	0.09			0.08			0.05		
% correctly classified	63.3			60.2			60.2		

Coefficients statistically significant at the 0.05 level or better are shown in bold

The interaction coefficients for the first two variables—the availability of jobs locally and the level of benefits—but not for the third—lower benefits would create a greater incentive for people to work—suggest patterns comparable to those discussed above for the other independent variables. Virtually all of the coefficients, even those that are statistically insignificant, are negative, with exponents less than 1.0. They indicate that, over time, the regional divide in attitudes has narrowed. Thus, for example, the positive coefficient of 0.60 for the Midlands on the first variable in Table 4 indicates that on average across the years when Thatcher was in office (i.e. pre-1990) respondents there were 82 per cent more likely (an exponent of 1.82) to consider there were plenty of jobs available locally than was the case in the Northern regions. But the four negative interaction coefficients for the Midlands region with each of the succeeding periods indicate that that gap narrowed, more so in the Blair than the Major years, and more so still in the Brown years, but then reopened somewhat in the final period of coalition government.^5^ The same sequence applies to the pattern of coefficients for London, the South, and Wales on that variable: during the Major, Blair and Brown governments the gap between respondents in the North and four other regions (Midlands, London, South, and Wales)—but not Scotland—in their adoption of anti-welfare attitudes narrowed, with some evidence that it widened again, but not to the same extent as in the Thatcher years, in the final period when Cameron led a coalition government with the Liberal Democrats. With a few exceptions, the same patterns occur with respondents’ views on benefit levels—over time the gap between the Northern regions and those further south in the proportion feeling that benefit levels were too high and discouraged work (holding constant the other—individual level—independent variables) narrowed. But—as assessed by the statistical significance of the findings—on the third variable (whether lower benefits would create a greater incentive to work) such a narrowing occurred only in the comparisons between the Northern regions and London.

## And how about political leanings?

A further set of models, rather than focus on individual characteristics and regional location in analysing changing patterns of attitudes, assessed the pattern of differences between supporters of the three main political parties.^6^ In these, nine of the independent variables included in the basic tests (Table 1) are excluded because they are significantly related to party choice. To avoid the potential confounding effects of that collinearity, only period was retained and a further variable added; respondents were asked which party, if any, they identified with and these were classified into: Conservative, Labour, Liberal Democrat, and Other/None/Don’t Know.^7^

Table 5 shows the results of those three binomial logistic regressions. The pattern of coefficients for time period are as in the other analyses. Those for party identification show that, across the full three decades, Labour supporters were significantly less likely to display anti-welfare attitudes than were Conservative supporters (exponents of 0.33, 0.23 and 0.33 respectively for the three dependent variables). Liberal Democrat identifiers were also less likely to be anti-welfare than their Conservative contemporaries, though the difference was smaller than with the Labour-Conservative comparison.

**Table 5:** Binomial logistic regressions of three anti-welfare indicators by political party supported, with interactions

	Jobs for unemployed			Level of benefits			Incentive to work		
	b	se	exp	b	se	exp	b	se	exp
Constant	**1.23**	0.33		4.41	0.39		4.64	0.34	
Government (comparator—Thatcher)									
Major	**–0.51**	0.05	0.60	**–0.22**	0.03	0.80	0.03	0.05	1.03
Blair	**0.60**	0.05	1.83	**0.53**	0.03	1.69	**0.64**	0.05	1.90
Brown	**0.59**	0.06	1.81	**0.99**	0.05	2.70	**1.00**	0.06	2.72
Cameron	**0.20**	0.05	1.22	**1.00**	0.04	2.73	**1.06**	0.05	2.88
Party affiliation (comparator—conservative)									
Labour	**–1.10**	0.08	0.33	**–1.49**	0.05	0.23	**–1.11**	0.09	0.33
Liberal democrat	**–0.82**	0.15	0.44	**–1.25**	0.17	0.29	**–1.07**	0.18	0.34
Other/none	**–0.38**	0.03	0.68	**–0.80**	0.02	0.45	**–0.58**	0.03	0.56
Interactions									
Major—labour	**0.20**	0.09	1.22	0.10	0.06	1.11	–0.01	0.10	1.00
Blair—labour	**0.46**	0.08	1.59	**0.49**	0.05	1.64	**0.25**	0.09	1.29
Brown—labour	**0.52**	0.11	1.68	**0.49**	0.09	1.63	**0.22**	0.11	1.25
Cameron—labour	**0.51**	0.09	1.66	**0.41**	0.07	1.51	**0.20**	0.10	1.22
Major—LibDem	0.14	0.16	1.15	**0.42**	0.18	1.51	0.28	0.19	1.32
Blair—LibDem	**0.26**	0.16	1.29	**0.49**	0.18	1.63	0.19	0.19	1.21
Brown—LibDem	0.17	0.19	1.18	0.32	0.21	1.37	–0.01	0.22	0.99
Cameron—LibDem	0.15	0.17	1.16	**0.44**	0.19	1.55	0.16	0.20	1.17
N	54,747			71,830			54,732		
Nagelkerke R^2^	0.11			0.13			0.09		
% correctly classified	63.2			62.6			62.7		

Statistically significant coefficients at the 0.05 level or better are shown in bold

The pattern identified by the interaction coefficients is similar to that shown in Tables 2 and 4, especially so with regard to the Labour-Conservative contrast. The positive coefficients shown for the Blair, Brown and Cameron years, when set against the negative coefficients for the all-period contrast, show that the gap between the two groups narrowed substantially, especially during the Blair and Brown Labour administrations. During those 13 years British citizens were more alike in their sharing of anti-welfare attitudes than they were previously and, to a much lesser extent, after (when the country had either a Conservative or a Conservative-led government respectively). There was less narrowing of the—albeit smaller—gap between Liberal Democrats and Conservatives, however.

The regression coefficients and their associated exponents clearly demonstrate the narrowing of the differences in anti-welfare attitudes between political party supporters, therefore, but neither the intensity nor the direction of that change is readily appreciated from those statistical parameters.

An alternative means of portraying the changes is given in Table 6, which uses the regression coefficients and exponents in Table 5 to estimate the probability that supporters of each of the three main parties agreed with the statement that there were plenty of local jobs available. The main shift was clearly among Labour supporters, who became substantially more anti-welfare, especially in the post-Major years, than supporters of either the Conservatives or the Liberal Democrats. The British population generally was becoming more right-wing in its attitudes and the three parties’ supporters were coalescing around an anti-welfare—or pro-workfare—position.

**Table 6:** The estimated probability of supporters of each political party agreeing that there are enough jobs available locally, by government period

Period	Thatcher	Major	Blair	Brown	Cameron
Party supported					
Conservative	0.77	0.67	0.86	0.86	0.81
Labour	0.53	0.46	0.77	0.78	0.70
Liberal democrat	0.60	0.51	0.78	0.76	0.68

## Discussion and conclusions

Three main conclusions emerge from these analyses. The first is that British citizens became substantially more anti-welfare and, implicitly, pro-workfare—or, in other terminologies, more right-wing or neo-liberal—over the last three decades, especially during the Labour administrations led by Blair and Brown. Secondly, this rightward move not only involved most groups within society (defined by age, trade union membership, occupational class etc.); in addition the attitudinal gaps within each of those groups narrowed during the Blair-Brown years, as did the gap between the regions. The country had become more homogeneously anti-welfare by 2010 than it had been during the Thatcher and, especially, Major administrations—with that gap only widening slightly under the Conservative-led post-2010 coalition government. Finally, the increase in anti-welfare attitudes was significantly greater among Labour than Conservative party supporters. By contrast, Bromley and Curtice (1999)—using BSA data—found no evidence that Blair’s ‘Third Way’ (Giddens 1998) had gained popular support, particularly among Labour voters. Our analysis suggests, however, that a shift towards neo-liberal values had started under Major and continued apace under Blair and Brown. Similarly, comparing data for 1996, 1998 and 2000, Hills (2001) found that after a convergence in attitudes between the first two dates, in 2000 there was again a wide gap between those Labour supporters who thought benefit levels for the unemployed were too high and those who thought them too low. Our findings therefore help to shed new light on relations between party policies, popular attitudes and their political impact but interestingly our findings appear to contradict ‘thermostatic’ expectations, to a degree. As governments have moved right over the past thirty years, public attitudes have failed to tack left.

In 2012, therefore, during a period of substantial unemployment (7.9 per cent of the economically active population—the highest since 1996), some 60 per cent of respondents to the British Social Attitudes survey claimed that people could finds jobs in their local area if they really wanted one, more than twice the figure only twenty years previously, when unemployment was even higher (at 9.8 per cent). This shift—commensurate with the attack on ‘shirkers’ in the government’s rhetoric after 2010—was paralleled by similar major changes in attitudes to the financial and other benefits available to the unemployed: there was a similar doubling in the percentage of respondents who believed benefits were too high and discouraged work, and in the percentage who thought that the benefit system’s generosity meant that people were too dependent on the state and insufficiently willing to ‘stand on their own two feet’.

These attitudinal shifts have not stimulated greater polarisation within British society, between supporters and opponents of a generous welfare state, however. Instead, they have been associated with a narrowing of the attitudinal gaps between most socio-demographic and -economic groups, as many of those with ‘left’ leaning and ‘liberal’ positions on such issues have moved considerably rightwards, towards those from whom their opinions were diametrically opposed only a few decades ago: those moves are reflected in the closing attitudinal gaps between the country’s regions and supporters of the main political parties. A major theme of the post-2010 coalition government’s rhetoric in promoting its austerity programme to counter the UK’s major budget deficit and national debt crisis following the banking crashes of 2008–2009 was ‘we’re all in it together’. Although the evidence is very clearly that neither the post-2008 recession nor the post-2010 austerity programme had an equal impact across all parts of society, nevertheless some elements of the rhetoric had been adopted, long before the events of 2008 and their aftermath. Britain had already become more homogeneous—and in many ways more right-wing—in its attitudes to welfarism.

Why should this be; why this very substantial shift—and why did it happen when it did, very rapidly, under the New Labour governments of 1997–2010? Such substantial ideological changes are unlikely to have been endogenous. Were they stimulated by those who rewrote the political agenda—notably Blair and others in their post-1994 promotion of New Labour; and if so, should the shift not have been much clearer, sooner, in the 1980s when an earlier generation of—Conservative—politicians also sought to change political attitudes? (Crewe—1988, 35—for example, concluded his study of attitudes in the 1980s that “the public has not been converted to Thatcherism—not to its priorities, not to its economic reasoning, not to its social values”.) Why did the changes not come about until the first ten years of New Labour governments—years of substantial economic growth and widespread prosperity (with significant redistribution of income, despite one leading Labour party politician indicating he was “intensely relaxed about people getting filthy rich … as long as they pay their taxes”; Hills 2014)? Did New Labour politicians lead public opinion and, if not (given the unlikelihood of such a major shift without some clear leadership), were the media the crucial animateurs of changed public opinion that the politicians responded to? And was the public more amenable at that time because the period of prosperity (initiated from 1994 under Major’s Conservative governments and sustained during the decade when Gordon Brown was Chancellor of the Exchequer) meant that fewer people than in the preceding three decades had made calls upon the unemployment and related aspects of the benefit system so that they were more susceptible to media claims that a small minority was unfairly benefiting from—if not actively defrauding—the benefit system?

By the first decade of the twenty-first century, other major changes in British society were also stimulating attitudinal changes—promulgated through not only the media but increasingly by political parties, to a considerable extent responding to claims made by the United Kingdom Independence Party regarding the impact of immigration, especially post-2004 unregulated immigration from within the European Union, on the British economy and welfare system. Access to social housing was regulated for much of the twentieth century by a waiting list procedure, for example, with only a relatively small number of urgent cases based on need allowed to ‘jump the queue’. But this was changed, as less social housing became available following the success of the ‘Right-to-Buy’ policy introduced by the Thatcher government in 1980, to a needs-based allocation system. Thus older people who had paid into ‘their’ welfare state—as they saw it—through the national insurance system and assumed that they and their children would be major beneficiaries, saw other groups—notably immigrants, many with large families—getting accelerated access: ‘their’ welfare state’s benefits—in access to schooling and the National Health Service as well as housing—were being denied to them and their families by immigrants and perceptions of this inequity fuelled their growing opposition to the welfare state and EU immigration dominating the ‘Brexit’ referendum debate. (For examples of this shift, see Dench et al. 2006; Kearns et al. 2014, have generalised Dench et al.’s argument by showing that attitudes to welfare in 2009 varied not only by socio-economic group but also neighbourhood context.)

Uncovering the mechanisms whereby the shift from welfare to workfare promoted by some politicians since the late 1970s has been adopted by the British population, particularly since the mid-1990s, and the relative role of economic prosperity, media portrayals of an underclass that has largely opted out of the workforce to live on benefits that are perceived as more generous than wages, is beyond the remit of this paper. The analyses reported here have identified a very substantial shift away from welfare and towards workfare that the Thatcher governments tried to initiate, which began to take effect under John Major but was achieved—and sustained—by the New Labour administrations of Blair and Brown and has not since been undermined by Conservative austerity, strongly anti-welfare policies that have been masked by the political rhetoric of social cohesion (cf. Ferragina and Arrigoni 2016). Society has converged on anti-welfare attitudes, reducing the spatial divide that has long characterised Great Britain and substantially altering the contest between the political parties.

## Footnotes

1 Such changes had long been debated within the Conservative party, as exemplified by a 1961 paper written by a future Chancellor of the Exchequer, Geoffrey Howe: see http://www.independent.co.uk/news/obituaries/geoffrey-howe-one-of-the-architects-of-the-thatcher-revolution-who-became-one-of-the-primary-factors-a6689811.html.

2 In his speech at the 2012 Conservative party conference, the Chancellor of the Exchequer, George Osborne, asked ‘Where is the fairness, we ask, for the shift-worker, leaving home in the dark hours of the early morning, who looks up at the closed blinds of their next-door neighbour sleeping off a life on benefits?’, although one of his colleagues claimed that similar language was used two years earlier by a Labour Cabinet Minister who claimed that ‘many people on the doorstep at the last election felt that too often we were for the shirkers, not workers’ (Jowit 2013).

3 Responses to the three variables were only very weakly correlated, so they were not combined into a single attitudes to welfare variable.

4 Given the large sample sizes, it is likely that even small differences would have statistically significant results—though in the great majority of those displayed here the differences were significant at the 0.001 level or better; the size of the coefficients is thus of more substantial importance than their statistical significance.

5 Although the coefficients and their associated exponents are substantially different from each other, use of the 2SE rules suggests that they are not statistically significantly different from each other, only from the situation in the Northern regions.

6 From 1983 to 1988 the separate Liberal and Social Democratic parties operated as an Alliance: they merged in 1988 to form, first, the Social and Liberal Democrat party—later shorted to Liberal Democrat. We use the latter term for those various combinations and names.

7 A multinomial logistic regression equation with those nine variables as the independents and party identification as the dependent had a Nagelkerke R^2^ of 0.15, with all sets of independent variables making a highly significant contribution (a probability of 0.001 or less) to the accounting for party choice.
